# Terahertz radiation and the skin: a review

**DOI:** 10.1117/1.JBO.26.4.043005

**Published:** 2021-02-13

**Authors:** Angelina I. Nikitkina, Polina Y. Bikmulina, Elvira R. Gafarova, Nastasia V. Kosheleva, Yuri M. Efremov, Evgeny A. Bezrukov, Denis V. Butnaru, Irina N. Dolganova, Nikita V. Chernomyrdin, Olga P. Cherkasova, Arsenii A. Gavdush, Peter S. Timashev

**Affiliations:** a Sechenov University, Institute for Regenerative Medicine, Moscow, Russia; b World-Class Research Center “Digital Biodesign and Personalized Healthcare,” Moscow, Russia; c Federal State Budgetary Scientific Institution “Institute of General Pathology and Pathophysiology,” Moscow, Russia; d Sechenov University, Institute for Urology and Reproductive Health, Moscow, Russia; e Russian Academy of Sciences, Institute of Solid State Physics, Chernogolovka, Russia; f Bauman Moscow State Technical University, Moscow, Russia; g Russian Academy of Sciences, Prokhorov General Physics Institute, Moscow, Russia; h Russian Academy of Sciences, Institute of Laser Physics of the Siberian Branch, Novosibirsk, Russia; i Novosibirsk State Technical University, Novosibirsk, Russia; j N. N. Semenov Institute of Chemical Physics, Department of Polymers and Composites, Moscow, Russia; k Lomonosov Moscow State University, Chemistry Department, Moscow, Russia

**Keywords:** terahertz technology, skin, terahertz spectroscopy and imaging, regenerative medicine, cancer detection and treatment

## Abstract

**Significance:** Terahertz (THz) radiation has demonstrated a great potential in biomedical applications over the past three decades, mainly due to its non-invasive and label-free nature. Among all biological specimens, skin tissue is an optimal sample for the application of THz-based methods because it allows for overcoming some intrinsic limitations of the technique, such as a small penetration depth (0.1 to 0.3 mm for the skin, on average).

**Aim:** We summarize the modern research results achieved when THz technology was applied to the skin, considering applications in both imaging/detection and treatment/modulation of the skin constituents.

**Approach:** We perform a review of literature and analyze the recent research achievements in THz applications for skin diagnosis and investigation.

**Results:** The reviewed results demonstrate the possibilities of THz spectroscopy and imaging, both pulsed and continuous, for diagnosis of skin melanoma and non-melanoma cancer, dysplasia, scars, and diabetic condition, mainly based on the analysis of THz optical properties. The possibility of modulating cell activity and treatment of various diseases by THz-wave exposure is shown as well.

**Conclusions:** The rapid development of THz technologies and the obtained research results for skin tissue highlight the potential of THz waves as a research and therapeutic instrument. The perspectives on the use of THz radiation are related to both non-invasive diagnostics and stimulation and control of different processes in a living skin tissue for regeneration and cancer treatment.

## 1 Introduction

The skin is the layer of flexible outer tissue covering the body and functions as an interface with the environment.^1^^,^^2^ The main function of the skin is the protection from external factors, for example, against various xenobiotics and pathogens. Water and temperature balances are also regulated based on the processes in the skin. The functions and dysfunctions of the skin, including many pathological conditions, have a significant impact on both the physical health and general wellness of a person.

A large number of past studies have greatly expanded our understanding of the structure and properties of the skin, and many tools for treatment and diagnostic purposes have been developed. A significant role in such studies is played by interdisciplinary, cutting-edge approaches coming from physics and biophysics. Among such approaches, the use of terahertz (THz) radiation looks particularly promising due to some recent achievements in the field. THz radiation is an electromagnetic wave with a frequency that lies in between the infrared and microwave regions—namely, in the 0.1- to 10-THz range ($1\;\mathrm{THz}=10^{12}\;\mathrm{Hz}$).^3^ Its wavelengths and photon energies range from 3 mm to 30  μm and from 0.41 to 41 meV, respectively. Due to the high sensitivity to biomolecules and water content and the low ionization of biological samples, THz-based methods have a great potential in biomedical research and diagnostics.^4^^,^^5^ Consequently, many efforts have been devoted to the development and application of THz methods in biomedical and biological fields.^5^^–^^7^

Some specifics of THz imaging and spectroscopy, as described below, make these techniques good candidates for the skin research. THz radiation is non-ionizing and is considered to be safe for humans at low powers. THz waves are strongly absorbed by water molecules, which limits their penetration into tissues by hundreds or even tens of microns. Thus, skin tissue is the ideal target for imaging using THz radiation due to its superficial location. The skin penetration is around 0.1 to 0.3 mm depending on the THz frequency.^8^ Both the content and state (free or bound) of water in tissue could be used as the markers for skin cancer detection and diagnosis of some other skin diseases. In addition to their diagnostic potential, THz technologies demonstrate perspectives for treatment using their effects on DNA demethylation and specific expression.

This review is divided into eight sections. Section 2 is devoted to the brief overview of the THz instrumentation. Section 3 provides information on the skin structure and properties. Section 4 overviews the recent THz imaging and spectroscopic techniques used for skin studies. Section 5 addresses the possible effects of THz radiation on skin cells and the extracellular matrix (ECM). Section 6 summarizes current achievements in the diagnosis and treatment of skin cancer. Section 7 covers some other recent perspective applications of THz technology in skin-related problems. Section 8 summarizes the reviewed material and addresses the limitations and perspectives of THz technology.

## 2 THz Instrumentation

A variety of techniques to generate and detect THz radiation have been developed in the past few decades, and these form the basis of the spectroscopic and imaging instruments.^9^ Among the existing schemes, two general types can be distinguished depending on the generated radiation—pulsed and continuous-wave (CW).

The most common CW-radiation sources are quantum cascade lasers,^10^^,^^11^ high-speed transistors, and diodes.^12^^,^^13^ Tunable CW THz waves may be obtained by backward-wave oscillators,^14^ parametric conversion,^15^ and photomixing and frequency multiplication.^16^ Broadband CW-radiation can be obtained as a part of the thermal source spectrum, such as that of mercury lamps and globars.^17^ The detection of CW-radiation is usually implemented by pyroelectric and optoacoustic (Golay cell) detectors ^18^ or by Li-He cooled bolometers.^19^ Here, we should also mention emerging THz-wave solid-state emitters and detectors, which are based on different two-dimensional materials, such as graphene and related heterostructures, as well as on novel physical principles of operation.^20^^–^^23^

**Fig. 1:**
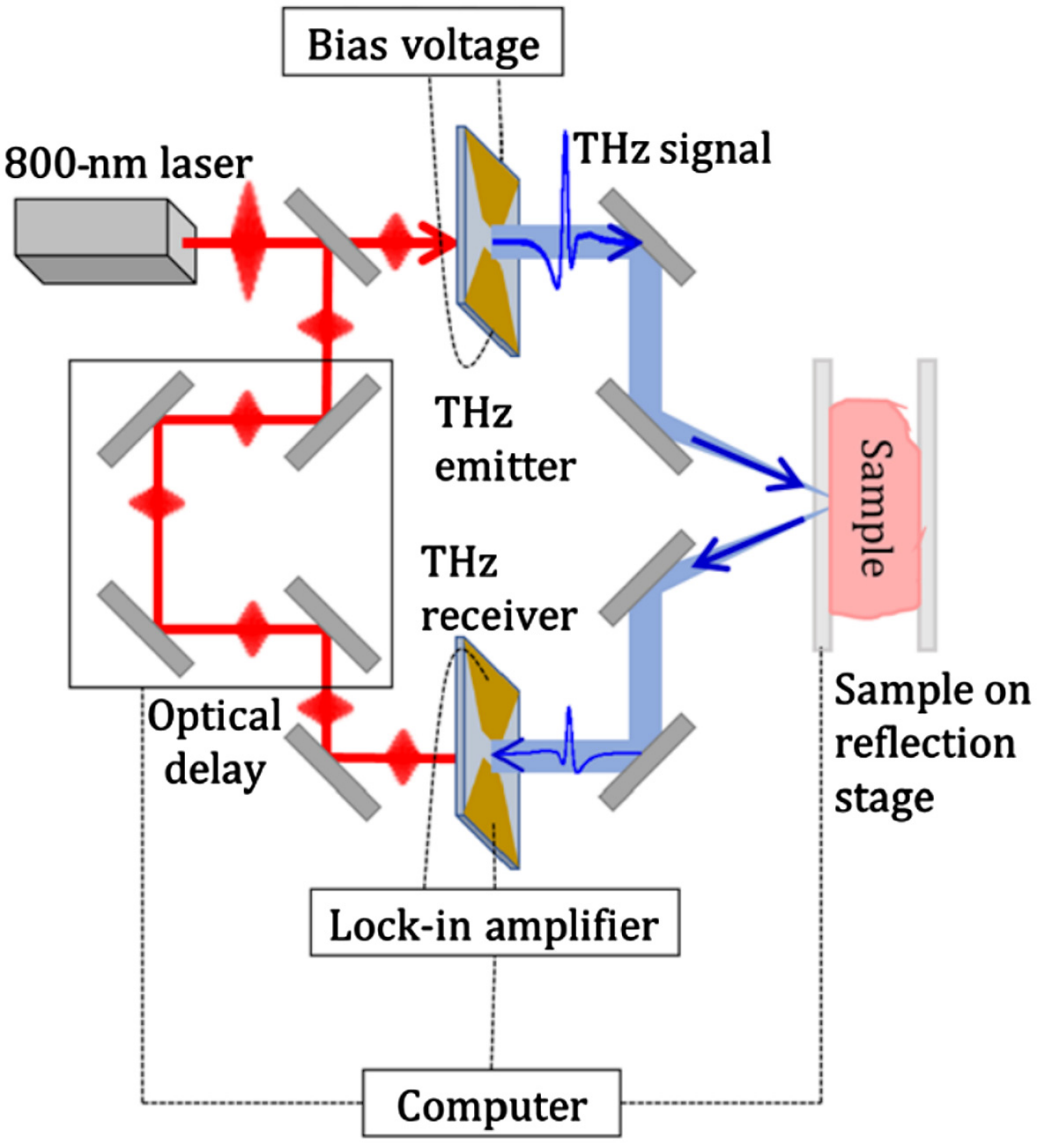
A common tissue measurement scheme for imaging and spectroscopy using TPS in the reflection mode (oblique incidence). The normal incidence and transmission mode are other widely used schemes. Reproduced from Ref. 24, CC BY-NC 4.0.

**Fig. 2:**
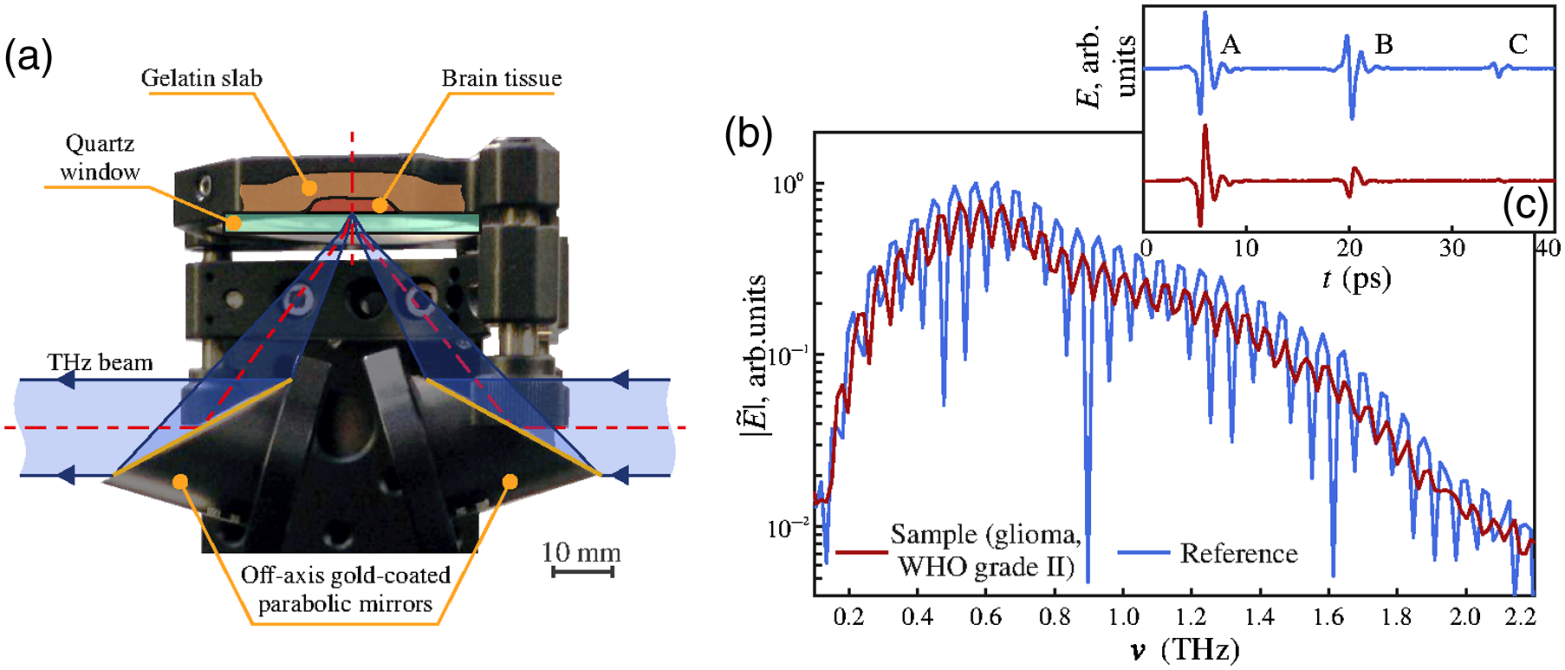
TPS of biotissue samples in reflection mode: (a) a spectroscopy unit for placing the sample; (b) reference $E_{r}$ without the sample and sample $E_{s}$ signals of the THz pulse spectrometer in the frequency domain; and (c) the same signals in the time domain. Reproduced from Ref. 32, CC BY 4.0.

The majority of studies on biological samples have been performed using THz pulsed radiation because it yields broader information than CW-radiation does (see below). The applied techniques are THz pulsed spectroscopy (TPS) and THz pulsed imaging (TPI); the schematic of a pulsed spectrometer is shown in Fig. 1. The pulsed radiation emitters are commonly based on photoconductive antennas (PCAs),^25^^–^^28^ although other methods exist, such as optical rectification and generation in plasma.^29^^–^^31^ Modern PCA emitters produce short sub-picosecond THz pulses, featuring only a few cycles of the THz field’s oscillation and a broadband spectrum. They are accompanied by femtosecond lasers, the pulsed “pump” beam of which proceeds to the PCA-emitter that generates a THz pulse due to the photoconductivity/photoswitching effect. In turn, a PCA detector is used for THz signal detection by mixing THz radiation and the “probe” beam of the femtosecond laser with an adjustable path length. The described THz-wave generation and detection principles underlie the methods of TPS and TPI. The typical forms of a THz pulse and its spectrum acquired from a biotissue sample are demonstrated in Fig. 2.

TPS might be implemented in reflection or transmission modes, and the imaging is generally achieved by raster-scanning a sample surface with a focused THz beam.^33^^–^^36^ At the same time, intensive research has been conducted on THz multipixel cameras and holographic or coded-aperture imaging principles.^9^^,^^37^^–^^39^

The main THz spectroscopic approaches that are generally used in biological research are Fourier transform spectroscopy (FTS), photomixing spectrometry, TPS, and TPI. FTS commonly utilizes broadband CW-sources or pulsed sources and the Michelson interferometer scheme, in which the inverse Fourier transform of the recorded interferogram is used. The photomixing spectrometer also utilizes a CW-source and contains two photomixers as a transmitter and a receiver, respectively.^40^^,^^41^ This technique is inexpensive and provides high spectral density and frequency resolution, although it requires a long measurement time. TPS is currently the most versatile technique in biological applications and is associated with the development of pulsed emitters and detectors.^5^ The technique allows for registration of the time-dependent electric field of a THz pulse (not just the power). The collected data include the amplitude and phase information in the frequency domain. Such a combination of time-domain and frequency-domain information about the THz field opens wide opportunities for TPS signal processing and data analysis, as compared with common Fourier-transform spectroscopy.

Data processing is an important step of THz spectroscopy, especially when using the TPS technology. Even when only the power spectrum is measured, the complex dielectric permittivity (or the complex refractive index) of the material can be obtained using the Kramers–Kronig relations and involving some additional assumptions.^42^^,^^43^ TPS does not require the use of Kramers–Kronig relations because both the frequency-domain amplitude and phase of the THz waveform are known. The data processing steps include preprocessing of raw signals, time-domain windowing (apodization),^44^^,^^45^ denoising ^46^^–^^48^ and then deconvolution (or inverse filtering),^47^ aimed at eliminating the impact of the particular TPS response function on the measured data. In TPS, reconstruction of the sample complex dielectric permittivity or complex refractive index is an ill-posed inverse spectroscopy problem related to the minimization of a discrepancy between the experimental data and the theoretical model. Various approaches for solving this inverse problem have been suggested and introduced recently for different geometries for experiments.^49^^–^^53^ Thr final processing steps often include statistical analysis and dimensionality reduction of the observed data.^5^^,^^54^

## 3 Structure and Properties of the Skin

The integumentary system is a protective barrier separating the body from the environment. It is represented by the skin and its derivatives. The skin is the largest organ of mammals, it accounts for about 16% of the body weight, and its total surface area reaches $2\;m^{2}$.^55^ It performs many vital functions, including thermoregulatory, metabolic, receptor, endocrine, and immune ones.^2^^,^^56^

**Fig. 3:**
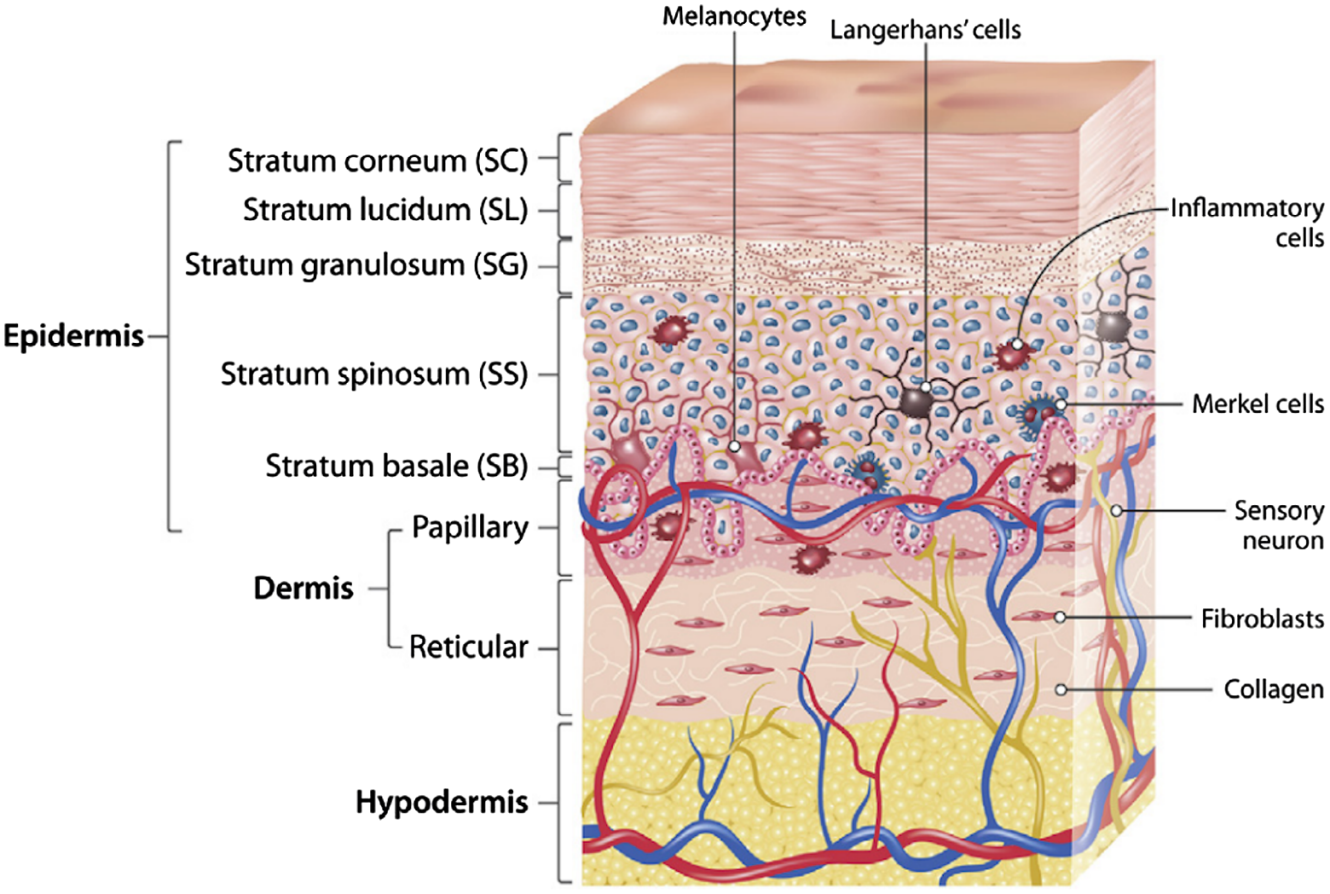
Schematic representation of the human skin structure. Reproduced from Ref. 57, CC BY 4.0.

There are three interconnected layers of tissues in the structure of the skin (Fig. 3). Its outermost layer is the epidermis, the middle one is the dermis, and the innermost one is the hypodermis.^58^ The complex dynamic organization of the skin is related to the different structure and physiological characteristics of different body areas.^59^ Depending on the localization, the thickness of these layers can vary.^60^^,^^61^ For example, the eyelid has the thinnest layer of the epidermis, less than 0.5 mm, while the palms and soles have the thickest layer of the epidermis, about 1.5 mm. The thickest dermis is on the back, where it is 30 to 40 times thicker than the overlying epidermis.^62^

The epidermis is a system of continuously renewing cells, which is based on the process of specific cell differentiation called keratinization. The morphological basis of the epidermis is the multilayer flat squamous epithelium. Its structural organization changes from the innermost basal layer to the outermost stratum corneum.^63^ Keratinocytes account for up to 95% of epidermal cells and enable the synthesis of keratin, a filamentous protein that plays a protective role.^64^ The epidermis is usually divided into four layers according to the morphology and position of keratinocytes:^65^

• the layer of basal cells (stratum basale);
• the layer of spinous cells (stratum spinosum);
• the layer of granule cells (stratum granulosum); and
• the layer of keratinized corneocytes (stratum corneum).

The basal layer is formed by one row of columnar keratinocytes adjacent to the basement membrane. The distinctive features of basal cells are dark-colored oval nuclei and the presence of the melanin pigment. Basal keratinocytes adhere to each other, as well as to more superficial flattened cells, through desmosomes.^66^ The presence of stem cells and the mitotic activity of the basal layer provide the continuous renewal of epithelial cells and their differentiation, with gradual lifting into overlying layers, transformation into corneocytes, and desquamation from the skin surface. The basal cell migration from the basal layer to the stratum corneum in humans takes at least 14 days, and the transit through the stratum corneum to the external epidermis requires another 14 days.^67^

The prickly layer (stratum spinosum) consists of several rows of large cells of a polygonal shape attached by desmosomes in the area of numerous processes (“spines”) that contain bundles of tonofilaments. Prickle keratinocytes retain the ability to reproduce through mitosis. The granular layer is formed by 1 to 3 rows of flattened spindle-shaped cells with a dark nucleus. The cytoplasm of such cells also contains tonofilaments, as well as keratohyalin granules. The clear layer is present only in the thick skin; its structure contains the eleidine protein. It consists of 1 to2 rows of flattened oxyphil cells with fuzzy borders and poorly defined organelles.^68^ The stratum corneum is the outermost layer of the epidermis, and it is formed by postcellular structures, corneocytes. They do not contain nuclei and organelles and are filled with keratin filaments (tonofilaments), which gives them high mechanical strength and resistance to chemicals. In the outer parts of the layer, desmosomes break apart, and corneocytes desquamate (shed) from the surface of the epithelium.^67^

The above-mentioned epidermal layers are involved in the formation of the epidermal proliferative unit. It is a self-renewing unit of the epidermis that has the shape of a hexagonal cell column. Its width is equal to the width of a single corneocyte, while its height corresponds to the thickness of the epidermis, and it includes all layers of the epidermis.^69^

In addition to keratinocytes, the epidermis contains populations of non-epidermal cells (melano-cytes, Langerhans cells, and Merkel cells). Melanocytes are specialized neuroglial pigment-synthesizing cells. Their body lies in the basal layer, and the long processes continue into the more superficial layers of the epidermis. Melanocytes produce melanin and transfer it to keratinocytes. Melanin is a black-brown or yellow-red pigment that protects the nuclear apparatus of cells from damage by ultraviolet rays. Melanin is synthesized and accumulated in melanosomes, which are transported to the processes of the melanocytes. The synthesis of melanin and its transport into epithelial cells is stimulated by melanocyte-stimulating hormone and adrenocorticotropic hormone, as well as by ultraviolet light.^70^

Merkel cells are neuroendocrine cells that are associated with the afferent nerve fiber and perform the receptor function. Their body lies in the basal layer, and the processes are attached to epithelial cells of the basal and prickly layers by desmosomes. In the basal part of the cell, granules that contain a mediator that is secreted into the synaptic cleft during the mechanical deformation of the processes are accumulated.^71^ Langerhans cells (or intraepidermal macrophages) are of bone marrow origin, and they lie in the basal or prickly layers. They capture antigens that penetrate the epidermis, process and transport them to the lymph nodes, and present them to lymphocytes, triggering an immune response.^72^

The dermis is the connective tissue layer of the skin that is about 0.5 to 5 mm thick and located under the epidermis. The dermis serves the trophic function, gives the skin strength, and contains its derivatives. It is composed of two layers—the papillary layer and the reticular layer. The papillary layer consists of a loose fibrous connective tissue with lymph and blood capillaries, nerve fibers, and endings. It provides the connection of the dermis with the basement membrane of the epidermis with the help of reticular fibers, elastic fibers, and special anchoring fibrils. The reticular layer of the dermis is deeper, thicker, and stronger: it is formed by dense fibrous unformed connective tissue and contains a three-dimensional network of thick bundles of collagen fibers interacting with the network of elastic fibers.^63^

The subcutaneous fatty tissue (hypodermis) is a continuation of the dermis. The structural basis of the skin’s deepest layer is the white adipose tissue and layers of loose fibrous connective tissue. The adipose tissue thickness depends on the location, gender, and nature of nutrition. For example, the hypodermis is absent in the vermillion border and eyelids, while the thinnest subcutaneous tissue is present in the neck. The subcutaneous tissue plays an important role in the body, acting as a heat insulator and a storage site for nutrients, hormones, and vitamins.^60^

As in any other tissue, proteins, fats, and carbohydrates form the basis of the organic composition of the skin. Proteomic skin studies have revealed that the skin contains from 155 to 174 different proteins. The main structural proteins are collagen type I, II, III, VI, XII, and XIV and other ECM proteins (elastin, lumican, mimecan, periostin, prolargin, decorin, and laminin), keratins, and cell proteins (desmoplakin, histones, actin, myosin, vimentin, and tubulin).^60^^,^^73^

Between the skin cells, there is an intercellular “cement,” which consists of polar and non-polar lipids.^74^^,^^75^ Lipids take part in the creation of the waterproof barrier and cell adhesion, as well as in the process of the cell desquamation. The lipid profile changes toward the surface of the epidermis. Quantitatively, ceramides account for the largest proportion (up to 50%) of the skin. They are followed by cholesterol (about 25%) and free fatty acids.^75^ Carbohydrates are mainly represented by mucopolysaccharides, glycogen, and glucose. Of inorganic substances, water makes up the largest proportion of the skin. The water content in the skin varies depending on a person’s age.^76^^,^^77^ The content of trace elements in the skin is low, 0.5%; the most common elements are copper, zinc, arsenic, and cobalt.

The boundary location of the skin implies its exposure to dangerous external factors.^78^ Wound healing is a complex sequential process including hemostasis, inflammation, proliferation, and regulation with the participation of cytokines.^79^ Violation of the normal biological response to skin damage resulting from an illness, injury, or surgery, as well as prolonged adverse effects, can lead to the development of complications. Understanding the molecular, cellular, and physiological mechanisms that govern wound healing is the key to the successful treatment of skin diseases.

**Fig. 4:**
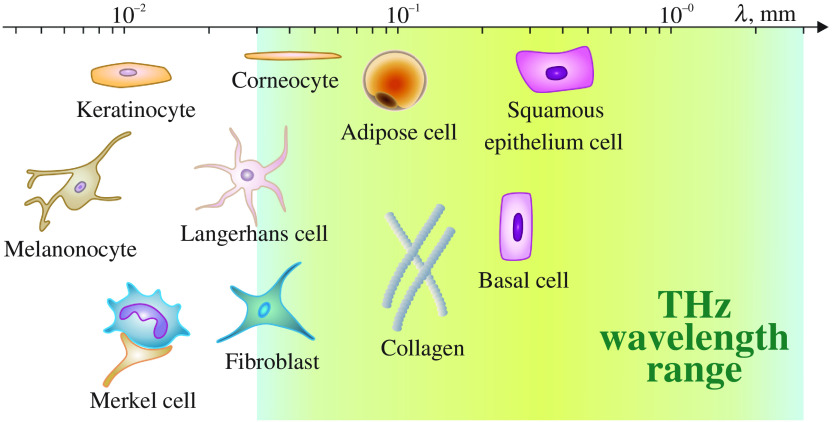
Comparison of typical skin components with the THz wavelength ranging from 0.03 to 3 mm. Courtesy of I. N. Dolganova.

Most of the skin components have dimensions of less than 0.1 mm, except for basal cells, squamous epithelium cells, and multicellular structures such as sweat ducts. Thus, they are much smaller than typical THz wavelengths. (Fig. 4). However, some other types of cells, such as adipose cells and thick bundles of collagen fibers, can cause scattering of THz waves, which should be accounted for during measurements.^4^

## 4 THz Spectroscopy and Imaging of the Skin

THz spectroscopy and imaging of biological tissues, including the skin, are based on the interaction of THz radiation with (predominantly) tissue water, as well as with other less-polar biomolecules, separated cells, and different structural components of tissues. The characteristic sample features are observed in the maximum or minimum values of the time-domain TPS waveform or in the frequency-domain data, such as the sample refractive index and absorption coefficient in a broad frequency range. The contrast-enhancement approaches, such as the integration technique,^80^ principal component analysis, linear discriminant analysis,^81^ or signal complexity analysis,^82^ are often applied to improve the capabilities of THz spectroscopy and imaging.

**Fig. 5:**
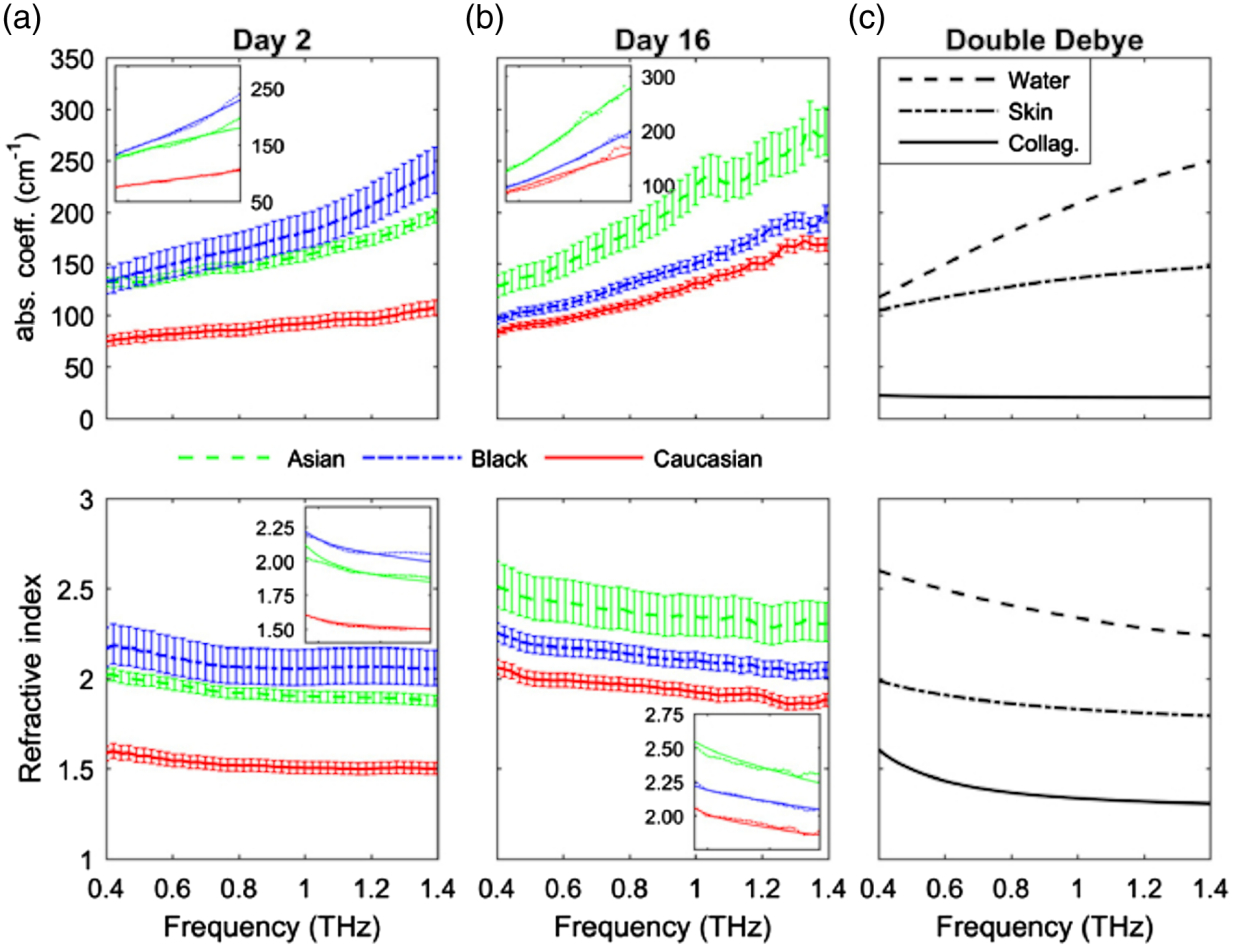
THz optical properties of Asian, Black, and Caucasian donor tissue models with a different content of melanin grown *in vitro* for 16 days: (a) measurements on Day 2, (b) on Day 16; (c) THz optical properties of water, skin, and collagen obtained from the double Debye model. Insets: fits from the double Debye model. Reproduced from Ref. 85, © 2019 Optical Society of America.

**Fig. 6:**
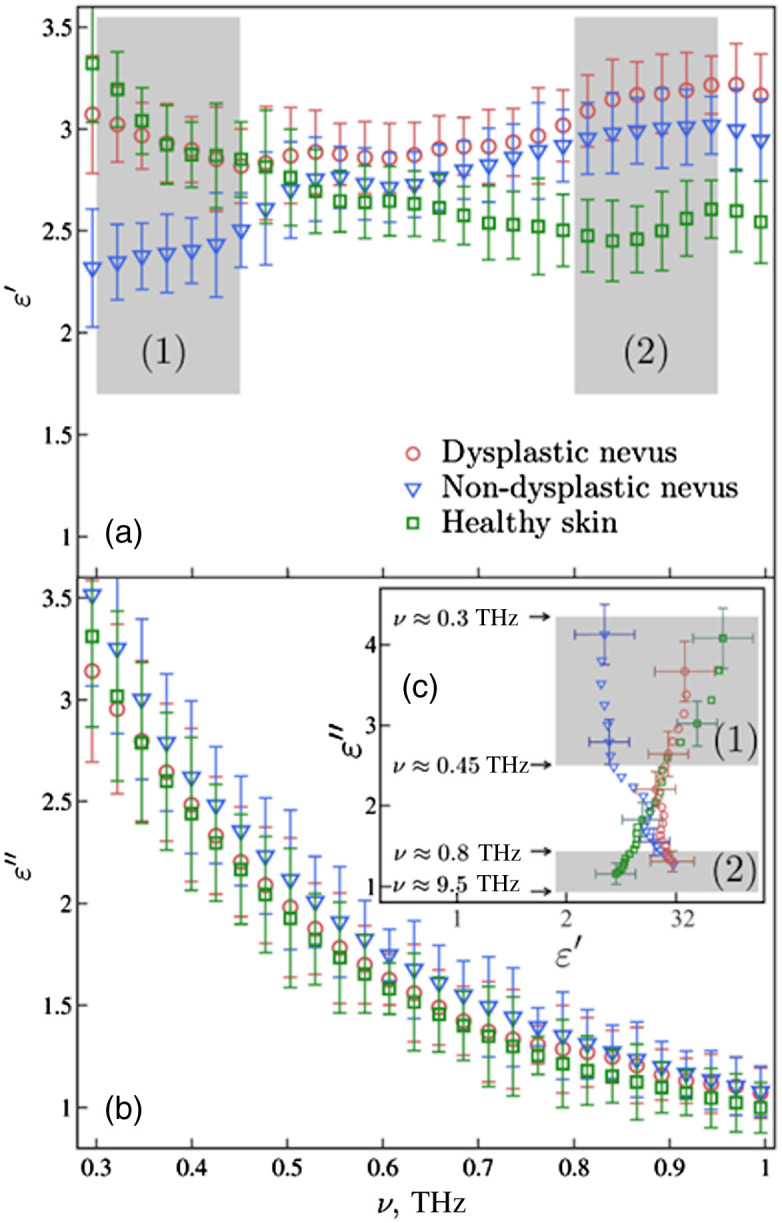
THz dielectric characteristics of healthy skin (in green), ordinary (in blue), and dysplastic (in red) nevi *in vivo*. Reproduced with permission from Ref. 86, © 2015 AIP Publishing.

THz spectroscopy was found to differentiate skin and muscle tissue with high sensitivity.^82^ TPS systems were successfully applied for measuring the optical properties of different skin regions.^83^ This method is sensitive enough to differ skin samples moisturized with glycerin or lanolin.^84^ The THz optical properties, such as the frequency-dependent absorption coefficient and refraction index, depend on the melanin content in the skin.^85^ Usually, the THz refractive index n of skin decreases with the frequency (see Figs. 5 and 6), though the opposite character was found for ordinary and dysplastic nevi^86^ (Fig. 6).

TPS allowed for detection of differences in the stratum corneum treated with chemicals that caused changes in the content of intracellular lipids or in the conformation of proteins.^87^ Free water content in the stratum corneum can be measured with THz spectroscopy.^88^ Also, it can detect the pressure changes in the skin due to different water distribution profiles ^89^ and the process of water desorption itself.^8^ The diffusivity of human skin can be measured and used for spatially resolved maps of water content in the skin.^90^^,^^91^ This information can be used for observing and investigating human scar healing.^92^ Reflection-mode TPI detected the water amount for a 7-h-long period after a skin burn.^93^ This method is also advantageous in collecting the data on a drug’s spreading within the skin. Additionally, the TPS and TPI methods appeared instrumental in studying dimethyl sulfoxide and glycerol diffusion into skin tissues,^94^^,^^95^ as well as related effects of tissue immersion optical clearing at THz frequencies.^96^^–^^98^

The important feature of skin imaging is the opportunity to detect, differentiate, and identify specific skin structures, such as glands, ECM, capillaries, and others. A combination of two tissue imaging modalities, optical coherence tomography and TPI, allowed for measurement of the diameter and THz dielectric properties of sweat ducts.^99^ Moreover, the properties of the sweat ducts were similar before and after the measurements. This indicates that THz imaging is safe for skin and therefore potentially can be used in the medical field. THz spectroscopy can detect changes in the spatial structure of the skin. For instance, the absorption coefficients for the 0.2- to 1.5-THz frequency band were shown to depend on varying concentrations of collagen (in the range of 2 to 3  mg/ml) and fibroblast densities (in the range of $10^{5}$ to $10^{6}\text{ cells}/\mathrm{ml}$) in the dermal equivalents.^100^

ECM is an important part of the skin that is responsible for cell growth and differentiation, tissue mechanics, and regeneration. One of the most pronounced components of the dermis is collagen. Collagen absorbs THz radiation, thus providing data about the tissue structure and enrolled processes.^101^ Far-infrared spectroscopy was able to detect the collagen amount and its spectral shape changes after the addition of a salt solution.^102^ TPS allowed for detection of different salt concentrations in the collagen layers and drug absorption in the skin.^94^^,^^102^ Using TPI, the concentration and spreading area of a drug released from a collagen–chitosan scaffold were assessed,^103^ and the protein–water dynamics during proteolysis of collagen-like substrates by a matrix metalloproteinase were studied.^104^ Some other components of the skin ECM, e.g. glycoproteins, can also be detected and used for the tissue structure mapping.^105^

## 5 Effects of THz Radiation on the Skin

The THz radiation impact on biological cells, tissues, and organisms have been of importance since the creation of THz sources, yet this remains insufficiently investigated. For example, the safety limits of the power density, which are set by The International Commission on Non-Ionizing Radiation Protection, are not established for frequencies above 300 GHz.^106^ The conducted studies, as reviewed in a number of articles,^107^^–^^109^ reveal that THz radiation can have both thermal (associated with the heating of the exposed object due to absorption) and non-thermal effects on biological objects. Like other electromagnetic waves, THz beams heat biotissues, and the extent of heating depends on the applied power, while the strong absorption by water represents an important factor related to the heating. The potential tissue heating with THz waves has been shown in several model studies.^110^^–^^112^ However, such heating is associated with CW radiation sources and high-power densities, whereas for pulsed sources the average power is generally too low to cause notable changes.

Another class of effects is related to non-thermal mechanisms of the THz-wave—biological system interactions. Supposedly, THz radiation can induce linear or non-linear resonance effects at the molecular level, specifically in deoxyribonucleic acid (DNA), in which local disruption of hydrogen bonds can further lead to modifications in gene expression.^4^^,^^108^^,^^113^ Thus, THz radiation can serve as a convenient and effective tool for cell activity modulation. On the one hand, the majority of the used THz intensities are not harmful to cells and do not cause any decrease in their viability.^114^ Studies performed on skin cells showed no signs of apoptosis and oxidative stress.^115^ On the other hand, there are data regarding THz-induced adipogenic differentiation of melanoma skin cancer MSCs and indications that THz can influence on the protein transcription.^116^^,^^117^ More detailed data are presented in Table 1.

**Table 1:** Effects of THz radiation on cells.

Frequency	Irradiance ($\mathrm{mW}/{\mathrm{cm}}^{2}$)	Exposure time	Object	Effects	Refs.
Broad spectrum centered at 10 THz	1	2, 6, 9 h	Mouse MSCs	Exposure of cells to THz radiation for 9 h caused changes in gene expression, whereas in response to shorter duration of exposure, the changes were less pronounced. The lipid inclusions that are a characteristic sign of MSC differentiation into adipocytes were clearly visible after 9 h of exposure.	Ref. 116
1) 2.52 THz;	1.2	1) 2 h;	Mouse MSCs	It was found that genes affected by prolonged irradiation are characteristic for already differentiated cells, i.e., for adipocytes, whereas genes differentially expressed after short (2 h) THz irradiation are characteristic of pluripotent stem cells.	Ref. 117
		2) 2, 12 h			
2) 10 THz					
1) 10 THz;	1.2	1) 2 h;	Mouse MSCs	1) The level of expression of the shock protein genes remains unchanged after 9 h of THz irradiation.	Ref. 114
		2) 9 h			
2) 2.52 THz					
				2) The level of the stress-responsive CRP gene that is activated in dying cells remains low in both the control and irradiated cells suggests the absent cellular stress response.	
0.14 THz	10, 30, 50, 70, and 100	20 min	hDF	120 h after the irradiation, the proliferative activity of the irradiated cells did not differ from the non-irradiated control. The level of NO production by irradiated fibroblasts did not differ from the NO level of non-irradiated cells. The 0.14-THz radiation of 10- to 100-mW power did not affect the functional activity of human skin fibroblasts.	Ref. 115
0.15 THz	0.4	20 min	hDF	1) No effect on cell cycle;	Ref. 118
				2) no effect on heat shock response;	
				3) increase in genome damage;	
				4) no effect on clastogenic genome damage;	
				5) no effect on telomere length; the THz radiation exposure *in vitro* caused non-thermal effects on the genome.	
2.52 THz	84.8	5, 10, 20, 40, or 80 min	hDF	Cellular temperatures increased by 3°C during all THz exposures. At the used power, radiation at 2.52 THz can generate thermal effects in mammalian cells.	Ref. 112
0.14 THz	10, 30, 50, 70, and 100	20 min	hDF	After exposure to THz radiation, the proliferative activity of the irradiated cells did not differ from the control. The level of NO production by irradiated fibroblasts did not differ from the control.	Ref. 115
0.38 and 2.52 THz	0.03 to 0.9	2 and 8 h	hDF, HaCaT cells	No DNA damage was found in HaCaT and hFB cells after irradiation.	Ref. 119
0.10 to 0.15 THz	0.4	20 min	hDF	The THz irradiation resulted in the genome damage in hDFs. No changes in the expression of proteins associated with DNA damage sensing and repair were detected, indicating that THz radiation exposure may affect genome integrity through aneugenic effects.	Ref. 118

The ECM component of the skin might be affected indirectly by the modification of cell activity with THz radiation. For example, wound healing was stimulated by TGF-β-induced synthesis of collagen after the irradiation.^120^ This finding implies a potentially beneficial application of THz radiation for skin regeneration.

## 6 Skin Cancer Detection and Therapy

**Fig. 7:**
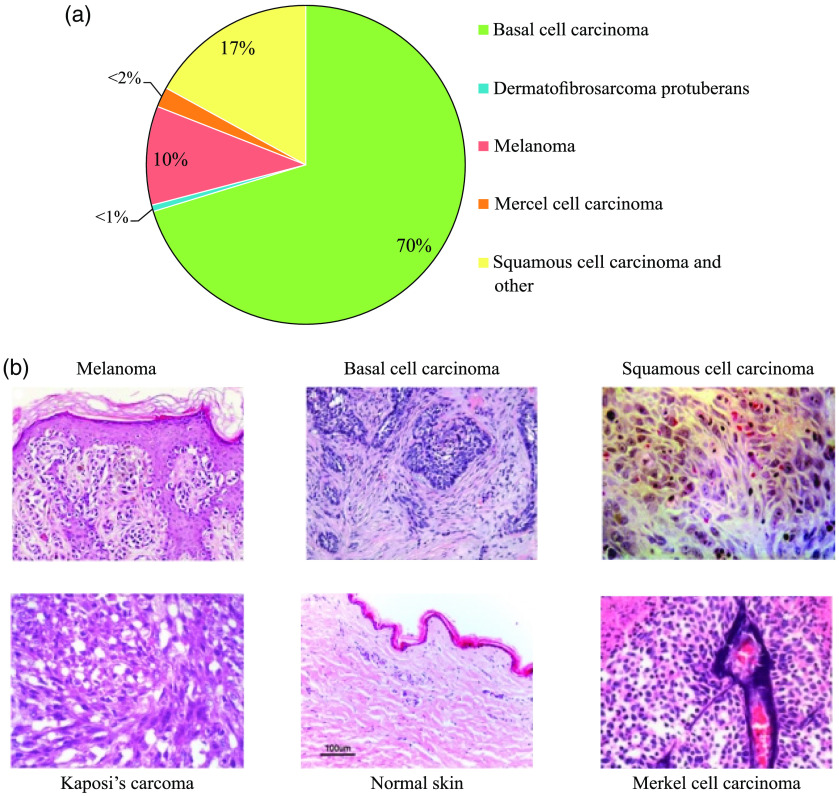
(a) Incidence of different types of skin cancer, reproduced from Ref. 123, CC BY 4.0. (b) Histological sections of melanoma, BCC, SCC, and some rare subtypes of skin cancer (Kaposi’s sarcoma and Merkel’s cell carcinoma). Reproduced from Ref. 124, CC BY-NC 3.0.

Skin cancer is the most common malignant disease in the world, affecting men and women of any race.^121^ Currently, one-third of all diagnosed types of cancer is skin cancer,^122^ and the incidence of skin cancer of all types is growing [Fig. 7(a)].

Skin cancer is generally divided into two main types: melanoma (reportedly, the most dangerous cancer of the skin) and non-melanoma skin cancer (NMSC).^125^ Melanoma develops from melanocytes that produce melanin, a pigment that stains the skin, eyes, and hair. Melanoma is one of the most aggressive and resistant to treatment types of human cancers, and it accounts for 75% of all deaths from skin cancer.^126^ NMSC is the fifth most common cancer, with more than 1 million diagnoses worldwide in 2018.^127^ NMSC is further divided into two types: basal cell carcinoma (BCC) and squamous cell carcinoma (SCC) [Fig. 7(B)]. Although BCC is the most common form of skin cancer, it is rarely fatal but can lead to serious health problems. SCC is the second most common form of skin cancer. Together, BCC and SCC make up approximately 95% of NMSC.^128^ In addition to the above-mentioned types, there are also some rare types of skin cancer, such as Merkel’s carcinoma,^129^ Kaposi’s sarcoma,^130^ and dermatofibrosarcoma protuberans (DFSP). Merkel’s carcinoma is the second leading cause of death from skin cancer after melanoma, although it causes less than 1% of malignant skin tumors.^131^ DFSP, which is ∼1.3 to 7.5 times less common than Merkel’s carcinoma, rarely metastases,^132^ and the prognosis is usually much better.

The development of new methods for the early-non-invasive and intraoperative diagnosis of skin cancer is extremely important. If skin cancer is diagnosed and treated early, it is almost a hundred percent treatable. A visual examination of the skin is usually not enough to diagnose it, and the traditional detection of histopathology (biopsy) is still the gold standard for evaluating skin cancer. However, biopsy has many disadvantages: it is painful, relatively expensive, and time-consuming and usually produces scars. In many cases, several biopsies are required to confirm the diagnosis. Therefore, non-invasive and minimally invasive methods and instruments are demanded.

**Fig. 8:**
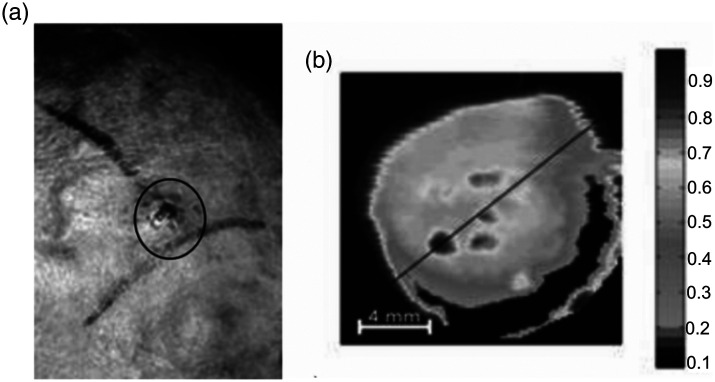
Basal cell skin cancer observed by THz imaging *in vivo*: (a) photograph of a skin sample and (b) a THz image of a skin sample, in which the central dark regions with increased absorption correspond to the tumor locus. Reproduced from Ref. 136 with permission, Copyright 2003 SPIE.

Currently, there are several methods for non-invasive diagnostics of epithelial tissues based on two- or three-dimensional skin imaging, including optical coherence tomography^133^^,^^134^ and confocal microscopy.^61^^,^^135^ For the first time, using a THz imaging system to visualize a malignant skin lesion was proven by Woodward et al.^136^^,^^137^ In that study, visualization of basal cell skin cancer was achieved due to the difference between the absorption coefficients of cancerous and normal tissues ^136^ (Fig. 8). Recent studies have shown that THz spectroscopy and/or spectroscopic imaging methods can identify tumors in mammary glands,^138^ lungs,^139^ the pancreas,^139^ and the brain.^32^^,^^140^

In Ref. 86, differentiation was demonstrated between ordinary and dysplastic human skin nevi *in vivo* using TPS, a dysplastic nevus being considered a precursor of melanoma, i.e., the zeroth stage of its development (see Fig. 6). For *ex vivo* murine skin tissues, the differences of optical properties between the normal skin and melanoma were used for selecting the borders of melanoma regions.^141^

The appearance of an oncological neoplasm leads to an increase in the blood microvasculature and, consequently, to an increase in the tissue water content. In addition to the structural changes, the content of various chemical compounds, for example, tryptophan amino acids,^142^ is altered in the affected areas of skin tissues. This leads to a modified spectral dependence of the reflection coefficient in the THz region, suggesting the possibility of label-free skin cancer diagnostics using reflected THz radiation.

TPI was applied to analyze the dielectric properties of human skin.^143^ Using this technique, a contrast was found between BCC and normal healthy skin. THz imaging was also utilized to analyze biological tissues using frequency conversion in gold nanoparticles and integrating an infrared camera and detector.^144^ Joseph et al.^145^ differentiated non-cancerous and cancerous tissue areas via two-frequency THz imaging at 1.39 and 1.63 THz.

Recently, a perspective application of THz radiation in skin cancer treatment was demonstrated. It was shown in Ref. 146 that expression of certain tumor suppressor genes can be regulated by non-thermal effects of intense THz radiation, which suggests that it can have an anti-cancer effect.^147^ Intense THz pulses downregulated the expression of epidermal differentiation genes, which are overexpressed in psoriasis and skin cancer.^146^ Non-thermal intense THz pulses with high (1.0  μJ) or low (0.1  μJ) energy applied for 10 min to human skin caused phosphorylation of H2AX, which indicates the formation of double-stranded breaks in DNA. The latter is extremely dangerous and can lead to cell death or cancer. However, the simultaneous activation of certain tumor suppressor proteins and regulatory cell cycle proteins, such as p53, p21, p16, and p27, which slow down the cell cycle and facilitate DNA repair, suggests that DNA damage caused by a THz pulse can be quickly restored.^148^^,^^149^

Methylation of genes that control the cell cycle and apoptosis catalyzed by DNA methyltransferases (DNMT) is a well-studied epigenetic change that causes genetic mutations leading to carcinogenesis.^150^ DNA demethylation has been shown to reduce the risk of cancer by preventing tumor suppressor hypermethylation or metastasis.^151^ Currently, there are some commercially available nucleoside inhibitors of DNMT, such as azacitidine (5aza, Vidaza^®^) and decitabine (5azadC, Dacogen™);^150^ however, such drugs have many side effects caused by their low specificity.^151^ Therefore, there is a need to look for alternative approaches to DNA demethylation. THz radiation can be used as an epigenetic inhibitor in the treatment of cancer due to its ability to cause targeted DNA demethylation, similar to demethylation drugs, along with an easy control of radiation energy.^152^ Demethylation is related to the resonant absorption of high-power THz radiation at the characteristic 1.65-THz peak associated with cancer cells and attributed to methyl-DNA bonds. Cheon et al.^152^ used resonant THz radiation to dissociate methyl-DNA bonds and reduce the total DNA methylation. The degree of methylation inside melanoma cells decreased by about 10% to 15%, causing the formation of 5 to 8 abasic sites [apurinic/apyrimidinic (AP) sites] per $10^{5}\;\mathrm{bp}$, which was significantly less compared with DNA damaged by infrared radiation.^152^ AP sites are one of the basic indicators of DNA damage, capable of generating DNA strand breaks, lethal mutations, or cell death.^153^ Therefore, it is necessary to understand whether powerful THz radiation can damage other DNA structures in addition to breaking methyl–DNA bonds.

Thermal effects of THz exposure are also applicable in cancer treatment, but they are less studied. Hyperthermic therapy is heating of tumor tissue (from 40°C to 45°C) to kill tumor cells.^154^ It is believed that the heat tolerance of normal cells is better than that of cancer cells due to a weaker blood flow and a worse cooling in tumor areas. Additionally, hyperthermia makes cancer cells more sensitive to radiation and drug therapy.

**Fig. 9:**
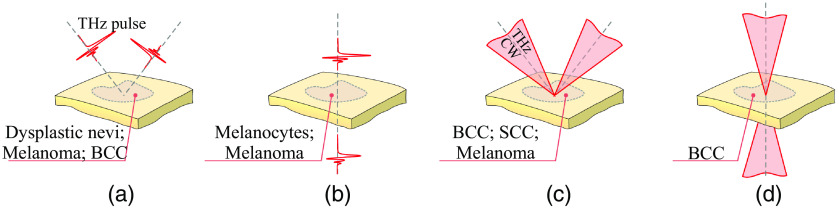
The possibilities of different THz techniques to detect skin cancer types: (a), (b) reflection and transmission mode TPI, respectively; (c), (d) reflection and transmission mode CW THz imaging and spectroscopy, respectively. Here, BCC stands for basal cell carcinoma and SCC stands for squamous cell carcinoma. Courtesy of I. N. Dolganova.

The current achievements of THz spectroscopy in the diagnosis and treatment of skin cancer are shown in Table 2 and in Fig. 9.

**Table 2:** The use of THz spectroscopy in the diagnosis and treatment of skin cancer (in the past 10 years).

Frequency	Description	Features	Instrumentation	Year	Refs.
1.39, 1.63 THz	The differences between normal skin and skin afflicted with NMSC were investigated.	Visualization was performed at two frequencies of 1.39 and 1.63 THz, which are within and outside the absorption band of tryptophan, respectively.	CW THz spectroscopy, transmitted signal	2010	Ref. 142
0.1 to 2.5 THz	The dielectric properties of human skin were analyzed to differentiate normal cells from abnormal cells (dysplastic and non-dysplastic skin nevi).	The results demonstrate that TPS is potentially an effective tool for non-invasive early diagnosis of dysplastic nevi and melanomas of the skin.	TPS, reflected signal	2015	Refs. 86 and 155
0.6 to 1.8 THz	Refractive index and absorption coefficient of melanoma were higher than those of normal tissue due to higher cell density and water content in tissue slices.	The melanoma was unambiguously identified in the frequency region of 0.6 to 1.8 THz.	TPS, transmitted signal	2020	Ref. 141
0.1 to 2.0 THz	Computational study on frozen normal skin and frozen melanoma images.	Ice is 100 times more permeable by THz radiation than liquid water, permitting imaging of frozen tissues to a depth of 5.0 mm and differentiating melanoma due to reflective boundaries.	Finite-difference time-domain computational modeling	2019	Ref. 156
0.2 to 1.4 THz	Visualization of dehydrated paraffin-embedded cutaneous malignant melanoma.	Methods of mathematical morphology and edge detection were applied to TPI images to distinguish normal and cancerous tissues.	TPI, reflected signal	2019	Ref. 157
0.3 to 1.0 THz	*In vivo* visualization of pigmented skin nevi and melanoma precursors inside healthy human skin tissues based on the analysis of THz dielectric constant curves.	Ability to differentiate dysplastic and non-dysplastic nevi using TPS was demonstrated.	TPS, reflected signal	2015	Ref. 155
0.4 to 1.6 THz	Melanoma samples had a higher refractive index and absorption coefficient than artificial normal skin in the studied frequency range; the contrast increased with frequency.	Static permittivity at low frequency and slow relaxation time can be reliable classifiers to differentiate melanoma from healthy skin regardless of the cell density.	TPS attenuated total reflection	2019	Ref. 158
4.2 THz	A technique for imaging human skin is suggested based on a THz-to-IR converter in combination with an IR-sensitive camera. The converter includes a system of gold nanoparticles embedded into a substance transparent in THz and IR (Teflon^®^).	The proposed technique laid a foundation for practical THz skin cancer imaging.	CW THz spectroscopy, THz-to-IR-converter	2017	Ref. 144
0.1 to 1.5 THz	Optical properties and spectral features of malignant skin melanocytes were investigated by TPS.	The difference in optical properties allowed for easy discrimination of mice’s malignant melanocytes from normal cells.	TPS, transmission mode	2020	Ref. 159
0.077 THz	A low-power 77-GHz CW radar design for biomedical imaging applications was proposed.	The radar can measure the dielectric properties of tissues, making it suitable for the detection of melanomas with an accuracy on the order of tens of microns.	Low-power multitone 77-GHz CW radar using a sensor based on low-cost Miniature Hybrid Microwave Integrated Circuit design	2020	Ref. 160
0.1 to 1.3 THz	Oral malignant melanoma was visualized using THz reflection imaging at room temperature and below the water freezing point (−20°C).	The images of the frozen tissue without liquid water showed better contrast due to the greater penetration of THz radiation into the sample. Significant structural differences between malignant oral melanoma cells and normal mucosal cells supposedly caused the contrast.	2-D and B-scan (reconstructed using the time-domain waveform) THz imaging	2013	Ref. 161
0.2 to 1.6 THz	A study on artificial human skin tissues with and without metastatic melanomas.	Both the refractive indices and absorption coefficients of the artificial skin with melanomas are higher than those of the normal artificial skin in the entire frequency range, due to a higher water content in malignant tissues.	TPS	2018	Ref. 162
0.584 THz	Study on thick excess cancer specimens, NMSC (BCC) and SCC.	Cross-polarized THz images exhibited lower reflectivity values in cancer as compared with normal tissue and allowed for correct identification of the tumor location.	Combination of polarized light imaging and CW-THz imaging (reflected signal)	2012	Ref. 163
1.39, 1.63 THz	Two-dimensional THz transmission images of non-melanoma skin cancers were acquired with a spatial resolution of 0.39 mm and compared with the histology data.	The difference in transmission between the normal and cancerous tissue was found to be 60% at both the used frequencies due to differences in water content.	CW-THz imaging (transmitted signal)	2011	Ref. 145
0.2 to 2.5 THz	Exposure of artificial human skin tissue to intense picosecond THz pulses affects the expression levels of numerous genes associated with non-melanoma skin cancers, primarily the genes of the epidermal differentiation complex.	The ability of intense THz pulses to cause favorable changes in the expression of multiple genes implicated in skin cancers was demonstrated.	THz pulse exposure	2013	Ref. 146
1.7 THz	The efficiency of DNA demethylation and damage were evaluated in melanoma cells based on the parameters of pulsed THz exposure.	The degree of methylation in the melanoma cell pellets decreased by approximately 10% to 15%, demonstrating the technique’s potential in cancer therapy.	THz pulse exposure	2019	Ref. 152
1.65 THz	DNA demethylation in melanoma cells using high-power THz radiation.	An effective non-invasive technique for melanoma therapy with few side effects.	Resonant high-power THz radiation	2020	Ref. 164

## 7 Other Application of THz in Skin Studies

**Fig. 10:**
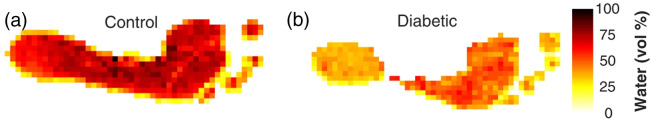
TPI of normal and diabetic feet and estimation of water content. Reproduced from Ref. 166, CC BY 4.0.

THz imaging is becoming one of the powerful tools for non-invasive diagnostics, visualizing and differentiating living, damaged, and dead tissue by changes in hydration gradients. For example, THz reflection imaging was proposed as a tool to monitor deterioration in the feet of diabetic patients. A common consequence of diabetes mellitus is the so-called “diabetic foot,” or “diabetic foot syndrome.” This syndrome is characterized by deterioration of micro- and macrovasculatue and innervation. This leads to an altered structure and physiology of the foot skin and underlying tissues with a decrease in skin sensitivity.^165^ Ultimately, it is often necessary to amputate a part of a leg or a limb as a whole.^102^ Early diagnosis is crucial for timely initiating the therapy and preventing the risks of ulceration, infection, and amputation. The existing approaches are based on the analysis of sensitivity and thermoregulation disorders and allow one to recognize the syndrome when innervation and vascularization are already violated. A new approach for early diagnosis of the diabetic foot using TPS based on the detection of a decrease in skin hydration was developed by a team of scientists from Mexico.^166^ Typical THz images of normal and diabetic feet demonstrated a correlation with the water content (Fig. 10). The TPS system used in this work utilized a reflection geometry for the generation of the feet images. A special platform was constructed with a chair and two high-density polyethylene windows, which were transparent at THz frequencies and used for the patients’ feet placement. The THz waveforms were collected across a mesh of 22×54  pixels spaced by 5 mm for each foot.

Visualization of burn wounds *in vivo* was achieved by correlating changes in the reflected TPS signal with a change in the local water concentration in soft tissues.^93^ The formation and dissipation of edema in and around the burn injury and the formation and evolution of the coagulation zone (highly reflective center of the burn) and a border zone of stasis (a ring of low reflecting tissue) were imaged with a high contrast in a live rat. In an other study,^167^ analyzing the changes in both the water content and the density of discrete scattering structures within the skin layers, the authors developed TPS-based approaches to non-invasively differentiate partial-thickness (second-degree) burns by the degree of damage. The deeper ones that require surgical intervention were distinguished from those that could naturally heal.

A reflective THz imaging system was used for visualization and quantification of the burn-induced model of edema in rats.^168^ 60×60  mm images were obtained in the reflective mode at the center frequency of 0.525 THz with a ∼125-GHz bandwidth, using a 0.5-mm step size per ∼10  min. The images and resulted data were compared with a more labor-intensive technique, depth-resolved magnetic resonance imaging, and a strong positive correlation was found. In another study,^92^ a reflection-mode TPS system was tested in scar imaging with the contrast based on changes in the refractive index and absorption coefficient of the scar; hypertrophic scars had a significantly higher refractive index than that of healthy skin, whereas normal scars had a refractive index lower than that of healthy skin. The refractive index in the scar area deviated from that of the surrounding healthy tissue even six months post-injury, which correlated well with the collagen deposition during wound healing.

The efficacy of the silicone gel sheeting strategy for skin scar repair was evaluated with THz spectroscopy.^169^ The mechanism of the silicone gel sheeting is not well established, and investigation of the skin water amount revealed the nature of the silicone sheets-induced regeneration.

Finally, a potential application of TPS is the analysis of archaeological findings and mummified tissues. Compared with traditional x-ray and computed tomography, TPS offers a lower spatial resolution but allows for better identification of bones and cartilage with a spatial resolution on the order of 1 mm (at 0.3 THz) that is limited due to wave diffraction. In addition, TPS can provide additional information on the optical density of the sample. By changing the used THz frequency, one can vary the level of details: lower frequencies (0.24 THz) revealed large vessels in the bone tissue, and higher frequencies (0.54 THz) visualized the morphology of the bone tissue itself.^170^

## 8 Conclusion

In this review, we summarized the recent developments of THz technologies related to the skin analysis, diagnosis, and treatment. The brief overview of instruments and methods demonstrates the uniqueness of information about skin tissue analyzed by THz imaging and spectroscopy. The research results obtained in the past few decades on THz-wave–biological tissue interactions highlight several directions for further studies, though a number of limitations slow down their implementation.

Fortunately, most of the limitations associated with the small penetration depth of THz radiation in biological tissues are less dramatic in the case of the skin. However, they might be important for the analysis of the deeper skin layers. Development of compact and inexpensive THz sources is another problem that limits the technique prevalence. The spatial resolution is defined by the diffraction limit and is not enough for single-cell detection. However, several promising techniques, such as THz solid-immersion microscopy,^35^^,^^36^ can overcome this limit and demonstrate high efficiency in biomedical imaging. The new effective approaches for signal enhancement, contrast and sensitivity improvement, and general signal analysis will be helpful for detection tasks. Nevertheless, the freedom from using contrast agents makes THz-based distinction between normal and pathologically altered skin most beneficial for *in vivo* applications.

The biological effects induced by THz radiation require further study. As shown above, the effects of low-power THz radiation are not harmful, but it is capable of inducing certain biological responses at the level of gene expression. This finding opens perspectives on THz radiation use in the stimulation and control of different processes in a living skin tissue related to regeneration of damages and cancer treatment. The impact of high-power THz radiation, as well as its possible application in the destruction of cancer cells, is less studied.

Thus, the recent advances in THz technologies used to study biological tissues and, particularly, skin tissues, reveal their further potential as research and therapeutic instruments.
